# Dissociation of Hydrogen and Formation of Water at the (010) and (111) Surfaces of Orthorhombic FeNbO_4_

**DOI:** 10.1002/cphc.202400781

**Published:** 2025-04-03

**Authors:** Xingyu Wang, David Santos‐Carballal, Nora H. de Leeuw

**Affiliations:** ^1^ School of Chemistry University of Leeds Leeds LS2 9JT United Kingdom; ^2^ Department of Earth Sciences Utrecht University 3584 CB Utrecht The Netherlands

**Keywords:** FeNbO_4_ surfaces, hydrogen dissociation, hydrogen diffusion and water formation

## Abstract

The orthorhombic structure of FeNbO_4_, where the Fe and Nb cations are distributed randomly over the octahedral 4c sites, has shown excellent promise as an anode material in solid oxide fuel cells. In this study, we have used calculations based on the density functional theory with a Hubbard Hamiltonian and long‐range dispersion corrections (DFT+U‐D2) to explore the adsorption and dissociation of H_2_ molecules and the formation reaction of water at the (010) and (111) surfaces. We have generated pristine surfaces with random distributions of cations from a 2×2×2 quasi‐random orthorhombic bulk structure. Specifically, we have considered various terminations for the (010) and (111) surfaces with different ratios of Fe and Nb cations in the exposed layers. The top and sub‐surface layers of the (010) surface move in opposite directions after relaxation, whereas the relaxed layers of the (111) surface shift outward by no more than 2.5 %. Simulations of the surface properties confirmed that the bandgaps are significantly reduced compared to the bulk material. We found that the hydrogen molecule prefers to dissociate at the O bridge sites of the (010) and (111) surfaces, especially where these are coordinated to Fe cations, thereby forming two hydroxyl groups. We have investigated the water formation reaction and found that the energy barriers for migration of the H ions are generally lower for the Fe/Nb−O sites than for the O−O site. Overall, our simulations predict that after dissociation, the H atoms tend to remain stable in the form of O_layer_‐H groups, whereas a larger barrier needs to be overcome to achieve the formation of water. Future work will focus on potential surface modifications to reduce further the barrier of migration of the dissociated H ions, especially from the oxygen bridge sites.

## Graphical abstract

DFT calculations demonstrate that at surfaces of the disordered orthorhombic FeNbO₄ material, H₂ molecules preferentially dissociate at Fe‐coordinated oxygen‐bridge sites compared to Nb‐coordinated sites, subsequently facilitating the formation of water molecules.

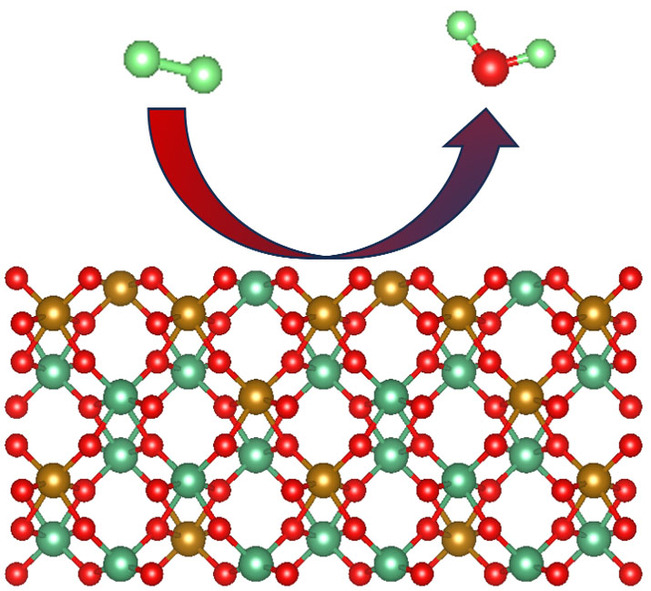

## 1 Introduction

Climate change and air pollution have become a global crisis that affects the whole of society. One way to reduce the risk of escalation is to deploy alternative green energy sources, like hydrogen, to replace traditional fossil fuel sources. Hydrogen is considered as one of the most promising sources to achieve a sustainable energy future, due to its abundant reserves in the form of water and its renewability.[^1^, ^2^, ^3^, ^4^, ^5^, ^6^, ^7^, ^8^, ^9^, ^10^, ^11^, ^12^]

Solid oxide fuel cells (SOFCs), which consist of solid‐state cathode, electrolyte and anode, are devices of particular interest, since they have high efficacy and the ability to process different fuels, including hydrogen, hydrocarbons and carbon monoxide, under medium and high temperatures (above 700 °C) to produce electricity.[^1^, ^2^, ^3^, ^4^] At the anode, reactions take place where fuel gases are oxidized to CO_2_ or H_2_O, providing electrons to the external circuit. Over the past years, Ni metal has been considered a good anode material, owing to its low cost, good catalytic ability and high electronic conductivity.[^5^, ^6^, ^7^] However, Ni‐metal anodes have shown poor tolerance to sulfur and carbon deposition,[^1^, ^3^, ^4^, ^6^] which limits the chemical activity under long‐term operation. As a result, several alternative ceramic materials, such as CeO_2_ and La_0.75_Sr_0.25_CrO_3_ (LSCr),[^6^, ^8^, ^9^, ^10^, ^11^, ^12^] have been developed to replace Ni metal as the anode material in SOFCs. Those oxide anode materials offer resistance to carbon deposition and stability during the operation of SOFCs at high temperatures, but they show lower catalytic efficiency and electronic conductivity than Ni‐metal anodes.^[6]^

**Figure 1:**
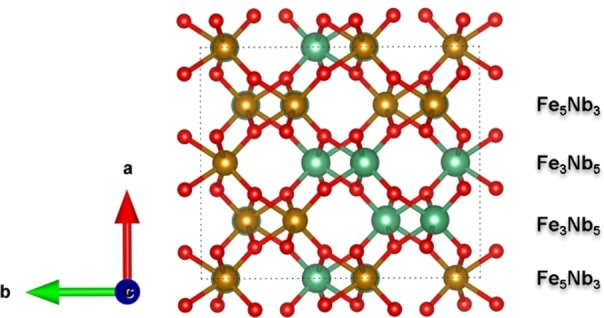
2×2×2 supercell of the orthorhombic FeNbO_4_ structure obtained from the ATAT model; O atoms are red; Fe atoms are yellow; Nb atoms are green.

In addition to the CeO_2_ and LSCr anode materials, research on Fe‐based materials, *i. e*. LaFeO_3_, FeVO_4_, and FeNbO_4_, has also made significant progress.[^13^, ^14^, ^15^, ^16^, ^17^, ^18^, ^19^] For example, previous studies have shown that orthorhombic FeNbO_4_ materials are good catalysts for hydrogen dissociation above 700 °C, where Nb^5+^ cations remain stable while Fe^3+^ cations are reduced to Fe^2+^ under an H_2_ atmosphere. In contrast to many other Fe‐based oxides, the Fe and Nb cations are distributed randomly in the orthorhombic structure (o‐FeNbO_4_) and thus layers consisting of only Fe^3+^ cations do not exist, see Figure 1. Furthermore, the random distribution of Fe^3+^‐Fe^2+^ chains throughout the whole material under operation ensures that electrons can be conducted along the 3D Fe−O−Fe network and continuous reduction of Fe^3+^ in the same layer can be avoided, which explains why the o‐FeNbO_4_ phase has good structural stability and relatively high electronic conductivity.[^14^, ^16^]

The reaction mechanisms for the oxidation of fuels, especially hydrogen, at the surfaces of o‐FeNbO_4_ is not as yet well understood. In this work, we have selected the (010) and (111) surfaces of o‐FeNbO_4_, which in previous have been shown to be the dominant surfaces,^[20]^ to explore their activity towards the H_2_ molecule. Random distributions of cations and different stacking sequences of the surfaces have been considered to simulate realistic disordered o‐FeNbO_4_ configurations. We have used calculations based on the density functional theory with a Hubbard Hamiltonian and long‐range dispersion corrections (DFT+U‐D2) to study the surface properties and reactions with the H_2_ molecule, including the dissociation of H_2_, diffusion of H atoms and formation of water at the surface.

## Computational Methods

The Vienna Ab initio Simulation Package, VASP (version 5.4.4), has been employed to carry out the DFT calculations.[^22^, ^23^, ^24^, ^25^] In all calculations, the ion‐electron interactions were described by the projector‐augmented wave method (PAW).^[26]^ The following electrons were treated as valence electrons: Fe (3p^6^3d^7^4s^1^), Nb (4p^6^5s^1^4d^4^4s^2^), O (2s^2^2p^4^) and H (1s^1^). We have employed the Perdew‐Burke‐Ernzerhof (PBE) generalized gradient approximation (GGA) functional to perform all spin‐polarized simulations.^[27]^ To enhance the accuracy of the description of the electronic structures, the on‐site Coulombic interaction (DFT+U) introduced by Dudarev et al.^[28]^ was chosen for the Fe 3d electrons with a U_eff_ value of 4.3 eV.[^29^, ^30^] The kinetic energy cutoff for the plane waves was set at 500 eV and all calculations were performed using the tetrahedron method with Blöchl corrections. The Henkelman algorithm was used to calculate Bader charges.^[31]^ A 2×2×2 supercell with random distributions of cations and a spin‐glass phase, comprising 16 Fe, 16 Nb and 64 O (Figure 1), was obtained via the special quasi‐random structures (SQS) method, which is based on a Monte Carlo simulated annealing loop with an objective function designed to perfectly match the maximum number of correlations.^[32]^ This method is implemented in the Alloy Theoretic Automated Toolkit (ATAT).[^33^, ^34^, ^35^] Specifically, Fe_5_Nb_3_, Fe_3_Nb_5_, Fe_3_Nb_5_ and Fe_5_Nb_3_ were distributed alternately along a direction in the 2×2×2 supercell. We have used the METADISE code^[36]^ to create the reconstructed dipolar (010) and (111) surfaces from the 2×2×2 supercell. The surface models consist of slabs of 8 layers along [010] and 4‐layer repeat units along [111], respectively, with 12 Å vacuum space in between the slabs and their images in neighbouring cells. 3×3×3, 2×2×1 and 3×3×3 gamma‐centered Monkhorst Pack grids were used for the simulations of the bulk, surface slabs, and isolated hydrogen/water molecules in a 10 Å×10 Å×10 Å cubic box, respectively. All calculations of the surface and bulk were carried out with the DFT‐D2 method implemented in VASP to incorporate the Van der Waals correction.^[37]^ The D2 parameters used were the pre‐determined C_6_ values of 10.80 J nm^6^ mol^−1^, 19.40 J nm^6^ mol^−1^, 5.71 J nm^6^ mol^−1^ and 0.14 J nm^6^ mol^−1^, and the R_0_ values of 1.485 Å, 1.908 Å, 1.342 Å and 1.001 Å were used for the Fe, Nb, O and H atoms, respectively. All geometry optimizations in this work were performed with the conjugate gradient method with a convergence criterion of 0.01 eV/Å. The scanning tunnelling microscopy (STM) images of the exposed surfaces were calculated using the Tersoff‐Hamman approach, where the STM tip approximates an infinitely small point source, as implemented in HIVE.^[38]^

In terms of the optimization of the surfaces, we fixed the bottom 50 % of atomic layers at their relaxed bulk positions, whereas the top 50 % were allowed to relax unconstrainedly to obtain the optimized surfaces. The Bader charges and magnetic moments of the surface atoms are calculated using an improved grid‐based algorithm and the work function is obtained using the following equation:

(1) $$\Phi =\;E_{vac}-\;E_{F}$$

where $E_{vac}$ is the vacuum level and $E_{F}$ is the Fermi level of the surfaces.

The Helmholtz free energy of the dissociation process of H_2_ was calculated using:

(2) $$\Delta F=\Delta E+\Delta E_{ZPE}-T\Delta S$$

where ΔE , $\Delta E_{ZPE}$ , and ΔS are the variation of the electronic energy obtained from the DFT calculations, the zero‐point energy correction, and the vibrational contribution to the entropy, respectively. We have calculated the vibrational frequencies through displacing by a short distance in the three Cartesian directions the adsorbed atoms only (H atoms and H_2_O molecules), and then obtaining the $\Delta E_{ZPE}$ and ΔS values via VASPKIT,^[39]^ which is a code that employs the harmonic approximation to calculate the entropy contribution to the free energy as:

(3) $$E_{ZPE}=\frac{1}{2}\sum_{i}\hbar \omega_{i}$$

(4) $$TS=RT\{\sum_{i}\frac{\frac{\hbar \omega_{i}}{k_{B}T}}{\exp\left(\frac{\hbar \omega_{i}}{k_{B}T}\right)-1}-\;\sum_{i}ln[1-\exp\left(-\frac{\hbar \omega_{i}}{k_{B}T}\right)]\}$$

where $\omega_{i}$ are the vibrational frequencies, R and $k_{B}$ are the gas constant and Boltzmann constant, respectively. The experimental entropy ΔS for gaseous H_2_ was obtained from the NIST database.^[41]^

The energy for the dissociation of H_2_ can be expressed as:

(5) $$\Delta E=E_{sur+H_{2}}-E_{sur}-E_{H_{2}}$$

where $E_{sur+H_{2}}$ represents the total energy of the surface with adsorbed dissociated hydrogen, $E_{sur}$ represents the total energy of the pristine surface and $E_{H_{2}}$ represents the energy of the isolated H_2_ molecule.

We employed the climbing image nudged elastic band (CI‐NEB) method to study the transition states of the surface migration of the H ions. To find the transition states, we inserted five consecutive images along the minimum energy path (MEP) from the dissociation of hydrogen to the formation of water to calculate saddle points, where the calculations were carried out using the limited memory Broyden Fletcher Goldfarb Shanno (LBFGS) method^[40]^ until the forces were smaller than 0.05 eV/Å. We confirmed that each transition state had an imaginary vibrational frequency along the reaction direction.

## 2 Results and Discussion

**Figure 2:**
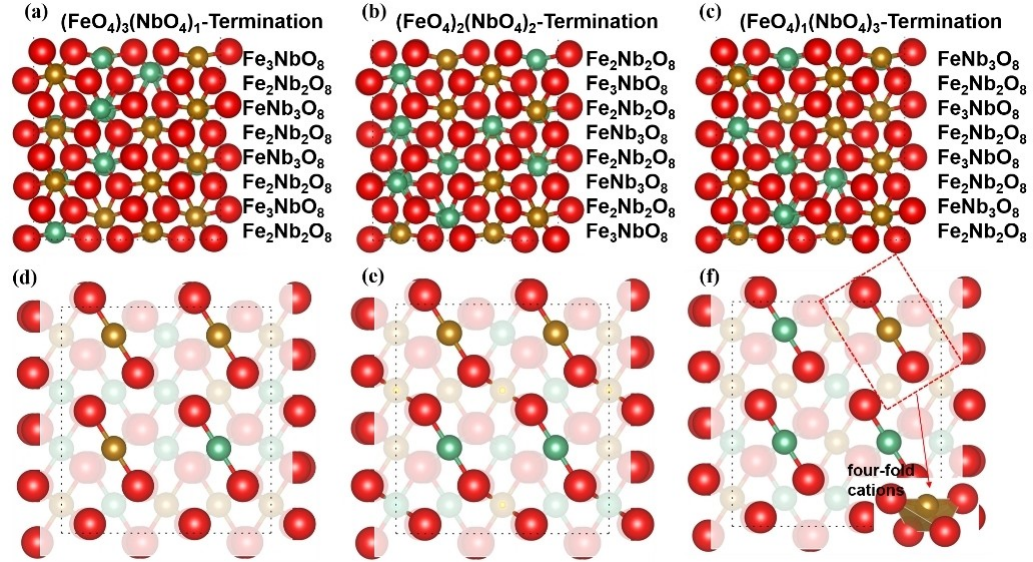
Side view (a–c) and top view (d–e) of the three terminations of the (010) surface and their corresponding stacking sequences; O atoms are red; Fe atoms are yellow; Nb atoms are green.

**Figure 3:**
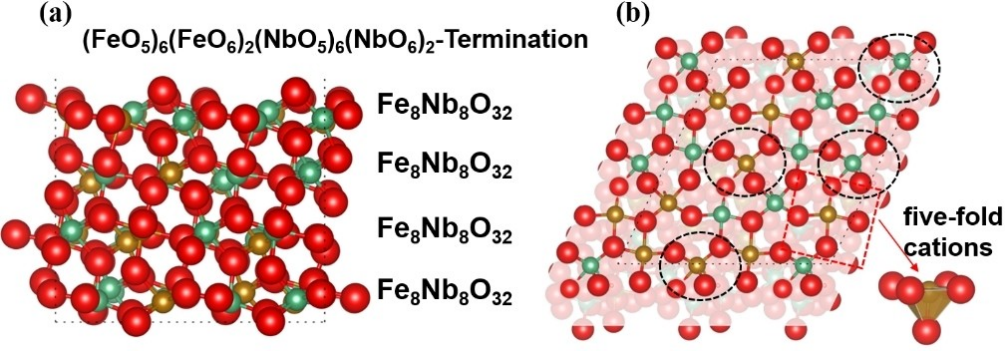
Side view (a) and top view (b) of the (111) surface and its stacking sequence; the black ellipse refers to the Fe/NbO_6_; O atoms are red; Fe atoms are yellow; Nb atoms are green.

Previous work[^42^, ^43^, ^44^] has shown, that the (010) and (111) surfaces are the two dominant surfaces of FeNbO_4_. In this work, we have therefore focused on these two surfaces and created simulation cells of 8 layers for the different (010) terminations and 4 repeat units for the (111) surface, see Figures 2 and 3. The stacking sequences in the (010) surfaces are −Fe_3_NbO_8_−Fe_2_Nb_2_O_8_−FeNb_3_O_8_−. Although this plane cannot be classified as a formal Tasker surface, our model is still valid to simulate the surface processes reactions. The Tasker type I (111) surface shows a stacking sequence of −Fe_8_Nb_8_O_32_−Fe_8_Nb_8_O_32_− along the z axis. In addition, we found that the exposed cations in the topmost layers of the (010) surfaces are coordinated by four oxygen atoms, forming two dangling bonds towards the vacuum. Thus, we have three terminations for the (010) surface, *i. e*. the (FeO_4_)_3_(NbO_4_)_1_, the (FeO_4_)_2_(NbO_4_)_2_ and the (FeO_4_)_1_(NbO_4_)_3_ terminations, which are henceforth called Fe_3_Nb_1_, Fe_2_Nb_2_ and Fe_1_Nb_3_ terminations, respectively, in the following discussion.

In contrast, each layer in the (111) surface comprises the same number of cations and we have therefore only explored the surface properties of one termination and its interactions with the H_2_ molecule. It is worth noting that in the top layers the two Fe and two Nb cations are fully coordinated by oxygen, whereas the other six Fe and six Nb are five‐fold coordinated cations, leading to one dangling bond each towards the vacuum, see Figure 3. From here on, we will denote the (FeO_5_)_6_(FeO_6_)_2_(NbO_5_)_6_(NbO_6_)_2_‐termination as Fe_8_Nb_8_.

### 2.1 Surface Properties

First, we have calculated the Bader charges, magnetic moments and work functions of the four surfaces, listed in Table 1, where the Bader charges and magnetic moments of the surface models are the average values of the exposed ions. Our simulations show that the Bader charge of the fully coordinated Fe cations in the bulk model is larger than that of the Fe cations with dangling bonds in the surfaces. Similar trends can be found for the Bader charges of the Nb and O ions, *i. e*. the Bader charges of the exposed ions are slightly smaller than those in the bulk structure. The magnetic moment of the Fe cations in the bulk is 4.21 μ_B_, which is reduced to 4.18, 4.16, 4.15 and 4.19 μ_B_ for the Fe_3_Nb_1_, Fe_2_Nb_2_, Fe_1_Nb_3_ and Fe_8_Nb_8_ surfaces, respectively. We found that the Nb and O ions in both the bulk and surface models have zero magnetic moments. The calculated work function of the Fe_1_Nb_3_‐terminated (010) surface is 7.29 eV, which is significantly larger than the other three surfaces.

**Table 1:** Average Bader charge (*q*) and the average magnetic moment (*m_s_*) of the ions on the top layers and the work function.

	*q*_Fe_ (e)	*q*_Nb_ (e)	*q*_O_ (e)	*m*_*s*Fe_ (μB)	*m*_*s*Nb_ (μB)	*m*_*s*O_ (μB)	Φ (eV)
Bulk	+1.84	+2.71	−1.14	4.21	0	0	–
Fe_3_Nb_1_‐Termination	+1.76	+2.62	−1.06	4.18	0	0	5.44
Fe_2_Nb_2_‐Termination	+1.75	+2.62	−1.06	4.16	0	0	5.13
Fe_1_Nb_3_‐Termination	+1.73	+2.59	−1.04	4.15	0	0	7.29
Fe_8_Nb_8_‐Termination	+1.79	+2.63	−1.03	4.19	0	0	6.02

In general, our simulations indicate that the Bader charges and the magnetic moments of the exposed ions are partially dependent on the coordination numbers. For instance, the five‐coordinated Fe and Nb cations in the (010) surfaces have larger values of *q* and *m_s_* than the four‐coordinated cations in the (111) surface, indicating that the five‐fold cations are in a higher oxidation state than their four‐fold counterparts. In addition, we found that the work functions of the (010) surfaces are generally smaller than that of the (111) surface, with the exception of the Fe_1_Nb_3_‐terminated (010) surface, suggesting that the number of exposed Nb cations can negatively affect the surface reactivity.

**Figure 4:**
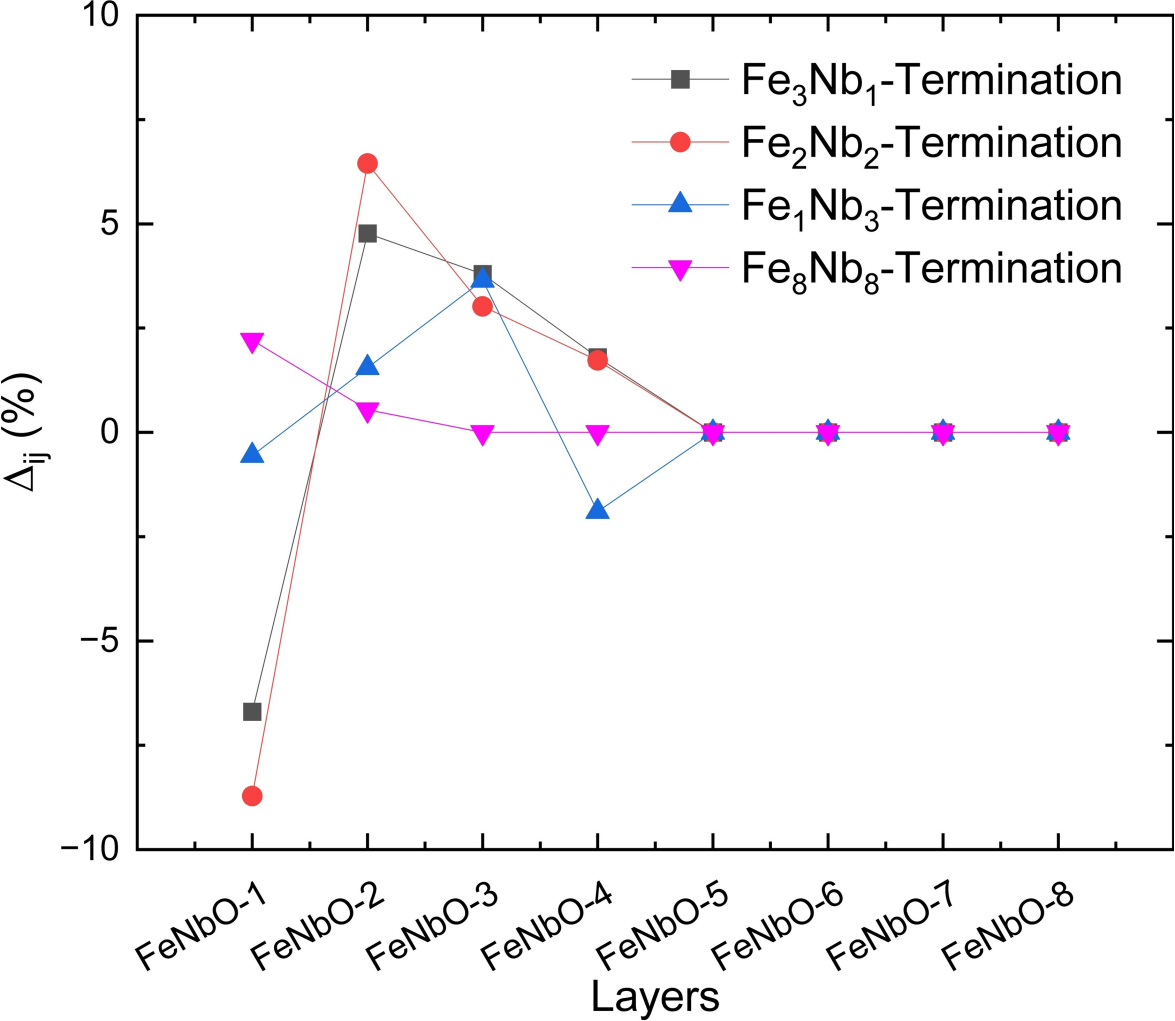
Interplanar relaxation of the four surfaces.

Figure 4 shows the interplanar relaxations of the three (010) and the (111) surfaces, which were defined as the difference between the atomic layers in the surface relative to their positions in the bulk structure. It can be calculated through 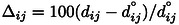 , where $d_{ij}$ and 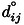 represent the separation between the relaxed *i*‐th and *j=i+1*‐th atomic layers in the surface model and in the bulk model, respectively. As mentioned above, the Fe_3_Nb_1_, Fe_2_Nb_2_ and Fe_1_Nb_3_ terminations contain eight layers, namely FeNbO‐1 to FeNbO‐8, whereas four layers constitute the (111) surface model and we have plotted the data from FeNbO‐1 to FeNbO‐4. Our simulations suggest that after relaxation the topmost layer FeNbO‐1 of the three (010) surfaces moves towards the bulk by 6.7 %, 8.71 % and 0.55 % in the Fe_3_Nb_1_, Fe_2_Nb_2_ and Fe_1_Nb_3_ surfaces, respectively. The subsequent layers from FeNbO‐2 to FeNbO‐4 experienced gradually decreased relaxation outwards from the bulk from 6.4–1.7 % and 5–0.5 % for the Fe_3_Nb_1_ and Fe_2_Nb_2_ terminations, respectively, whereas the Fe_1_Nb_3_ termination suffered the maximum relaxation of 3.6 % outwards from the bulk in the FeNbO‐3 layer. For the Fe_8_Nb_8_ surface we found that the topmost FeNbO‐1 layer shifts outwards by 2.5 % which trend decreased to 0.55 % for the sub‐FeNbO‐2 layer.

Overall, our calculations indicate that the relaxations of the FeNbO‐1 and FeNbO‐2 layers oppose to each other and thus, those two layers tend to merge to form the exposed surface. In contrast, we found that both the two relaxed layers of the (111) surface move outward from the bulk by no more than 2.5 %.

**Figure 5:**
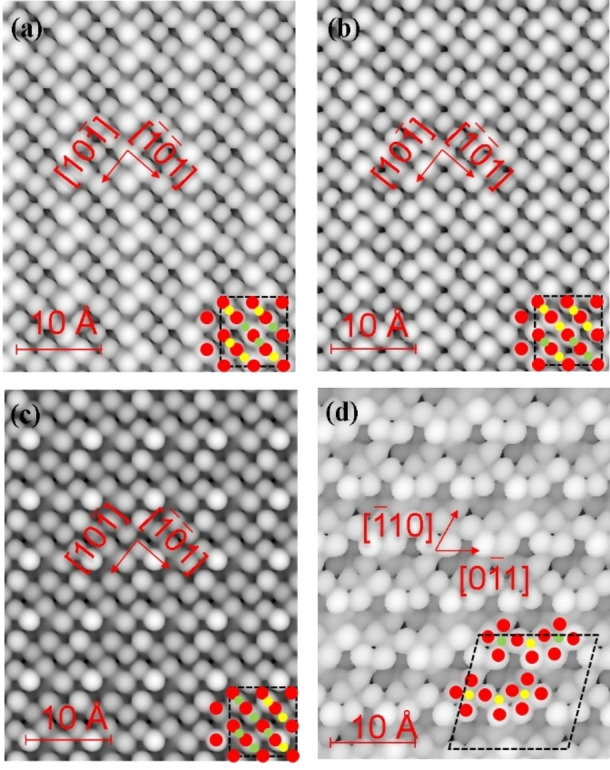
Scanning tunneling microscopy (STM) images of (a‐c) the Fe_3_Nb_1_‐, Fe_2_Nb_2_‐ and Fe_1_Nb_3_‐terminated (010) surfaces, respectively; (d) the Fe_8_Nb_8_‐termination of the (111) surface; O atoms are in red; Fe atoms are in yellow; Nb atoms are in green.

Next, we generated STM images at a bias of −3.5 eV, which are plotted in Figure 5. We found that the brightest spots represent the oxygen atoms, whereas the cations cannot be identified very well owing to their low resolution. The presence of cations from the sub‐surface layers can be seen on the (010) surfaces, in agreement with the analysis of the interplanar relaxation. In addition, our simulations suggest that the array of O−Fe/Nb−O−Fe/Nb appears along the $\left[\bar{1}0\bar{1}\right]$ direction in the (010) surfaces, and similarly the five‐fold Fe/Nb cations are distributed along the $\left[\bar{1}10\right]$ direction in the (111) surface.

**Figure 6:**
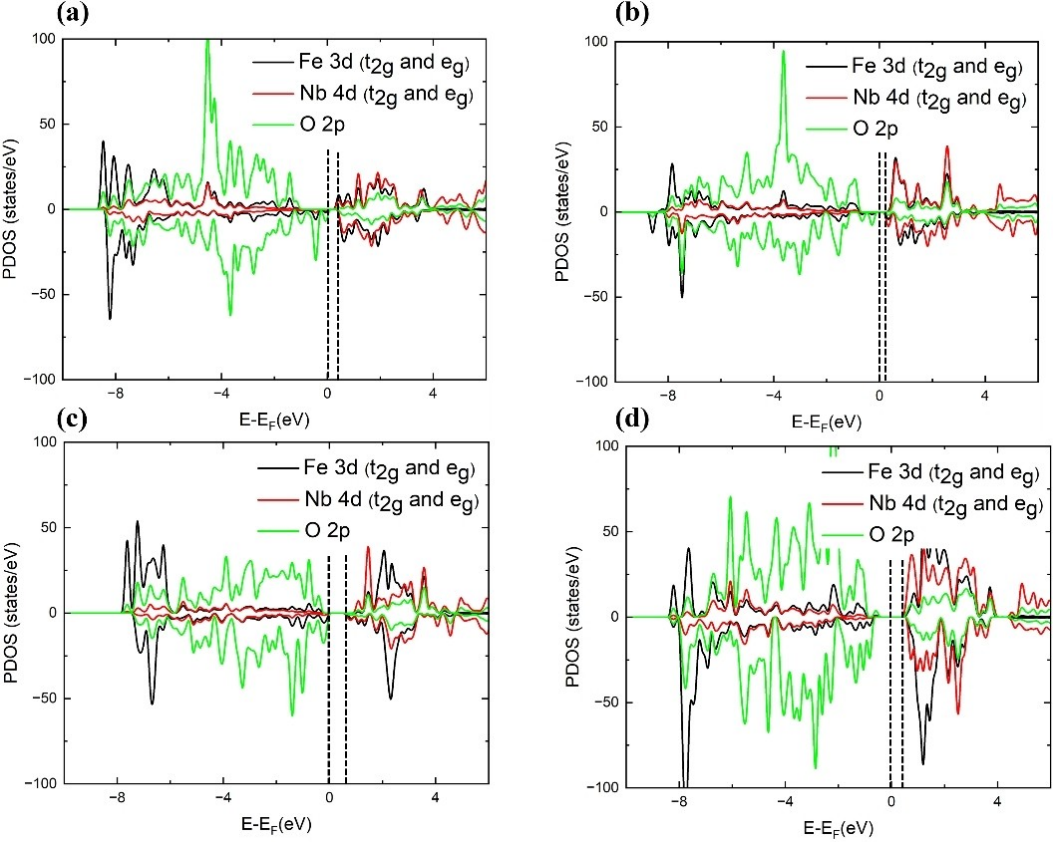
Projected density of states (PDOS) of the (a–c) Fe_3_Nb_1_‐, Fe_2_Nb_2_‐ and Fe_1_Nb_3_‐terminated (010) surfaces, respectively; (d) Fe_8_Nb_8_‐terminated (111) surface.

Finally, we have simulated the projected densities of states (PDOS) of the four surfaces, see Figure 6. The localized t_2g_ and e_g_ orbitals of the Fe ions near −8 eV are distributed over both the major and minor spin channels on the four surfaces, whereas the 3d states of Nb and 2p states of O are delocalized in the wide valence region from −8 to 0 eV. The unoccupied conduction region is mainly composed by the d states of the metal cations at those surfaces. In addition, our simulations indicate that in the (010) surfaces, the band gap decreases with the numbers of exposed four‐fold Fe cations, suggesting the sequence: $E_{bandgap}^{Fe3Nb1}<E_{bandgap}^{Fe2Nb2}<E_{bandgap}^{Fe1Nb3}$ . In general, we found that the band gaps in the surfaces are significantly reduced compared to the bulk material,^[20]^ because the existing metal‐oxygen dangling bonds on the exposed surface layers can behave as chemically active sites, as was also found in other studies.[^44^, ^45^, ^46^, ^47^, ^48^, ^49^]

### 2.2 Dissociation of H_2_

In our previous study,^[20]^ we found that the (010), (110), and (011) surfaces have similar exposed reaction sites. In this work, we have therefore focused on the (010) and (111) surfaces, including a number of distinct surface sites owing to the cation disorder in the orthorhombic FeNbO_4_ phase, to explore the catalytic reactions of hydrogen molecules at the surface. In a real disordered orthorhombic FeNbO_4_ structure, it is believed that the dissociation reactions of hydrogen take place at the surfaces, where Fe and Nb cations are distributed randomly. In this work, the four generated surfaces display random distributions of cations on a 2×2 scale. To explore the mechanisms of the surface reactions, we have only introduced one hydrogen molecule and placed it at different sites to investigate how the surface ions affect the dissociation reactions. We have classified two types of reaction sites for the dissociation of H_2_ and the formation of H_2_O on the (010) and (111) surfaces. One is defined as an Fe/Nb−O site which involves one cation site and its nearest oxygen site. The other one is an oxygen‐oxygen site comprising two adjacent oxygen atoms, each coordinated by two cations, and is therefore labeled as O_A1B1‐A2B2_, where the subscript A1B1 and A2B2 represents the cations to which the oxygens are bonded.

#### 2.2.1 (010) Surface

**Figure 7:**
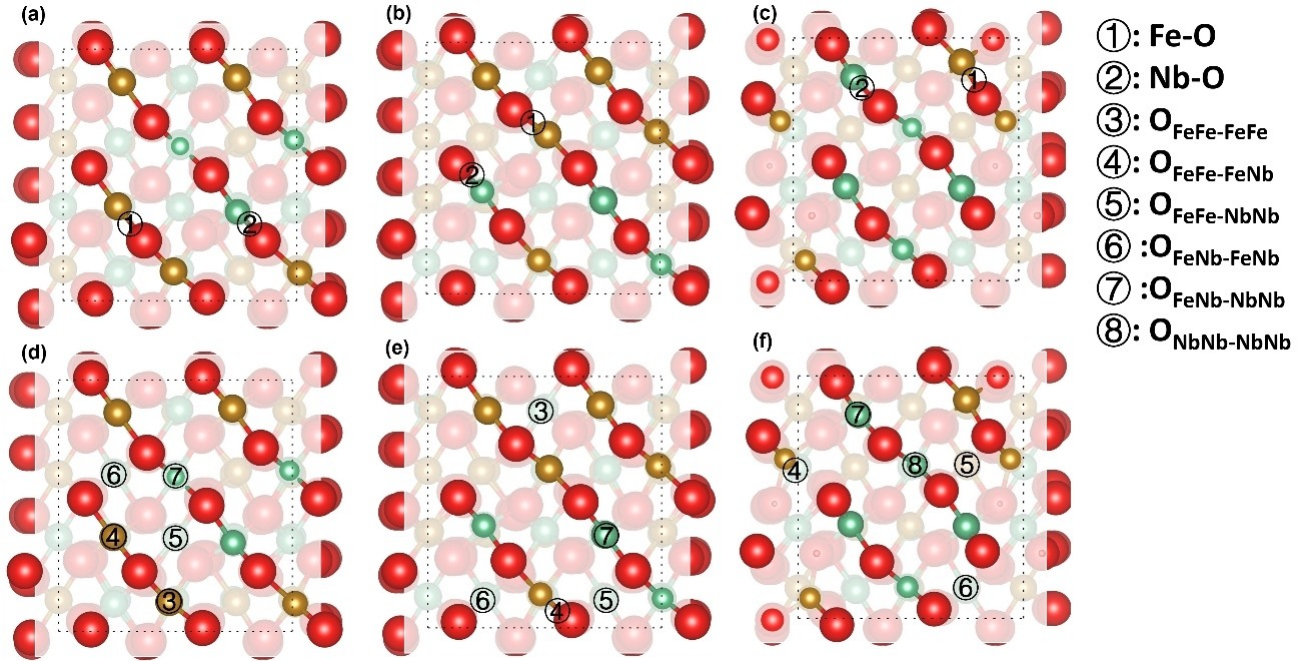
Top view of the Fe/Nb−O dissociation sites (a‐c) and O−O sites (d–f) of the (010) surfaces; (a, d) Fe_3_Nb_1_‐termination; (b, e) Fe_2_Nb_2_‐termination; (c, f) Fe_1_Nb_3_‐termination; O atoms are red; Fe atoms are yellow; Nb atoms are green, shading is used to enhance the visibility of the top atomic layers.

As shown in Figure 7, the Fe/Nb cations are coordinated by four oxygen ions in the exposed layers of the (010) surfaces. We identified three types of surface moieties, *i. e*. Fe−O−Fe, Fe−O−Nb and Nb−O−Nb on the (010) surfaces, creating two metal‐oxygen and six oxygen‐oxygen dissociation sites, which are represented by Fe−O, Nb−O $O_{FeFe-FeFe}$ , $O_{FeFe-FeNb}$ , $O_{FeFe-NbNb}$ , $O_{FeNb-FeNb}$ , $O_{NbNb-FeNb}$ and $O_{NbNb-NbNb}$ , respectively, see Figure 7. It is worth noting that we did not consider the $O_{NbNb-NbNb}$ , $O_{NbNb-NbNb}$ , and $O_{FeFe-FeFe}$ sites on the Fe_3_Nb_1_ Fe_2_Nb_2_ and Fe_1_Nb_3_ terminations, respectively, owing to the lack of Nb and Fe cations in the top and sub‐surface layers.

**Figure 8:**
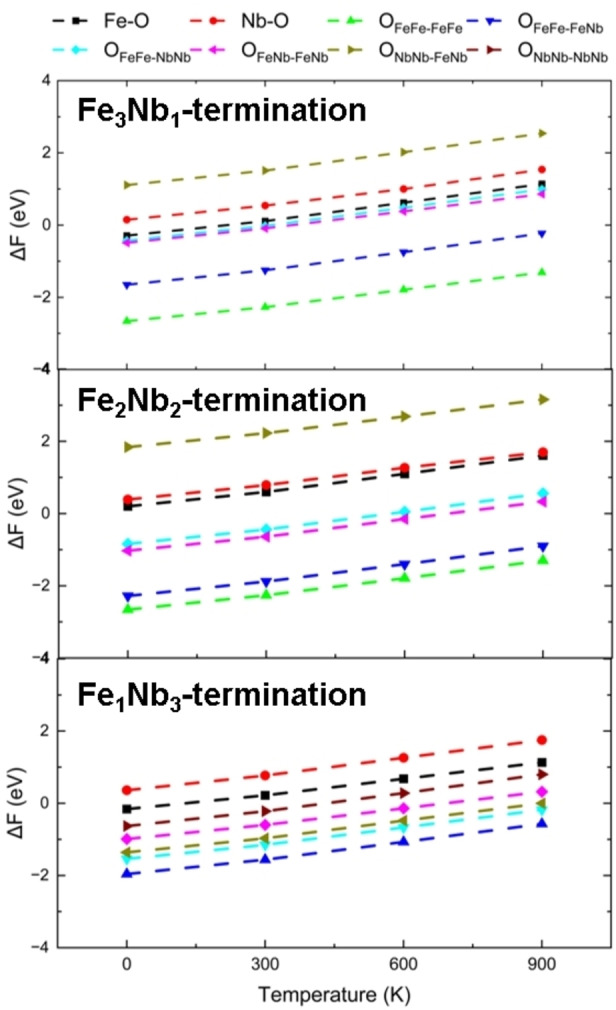
Dissociation energy of H_2_ on the (010) surfaces from 0–900 K.

First, we have calculated the dissociation energies at all sites on the surfaces from 0–900 K, see Figure 8. Our simulations show that the dissociation of H_2_ at the $O_{FeFe-FeFe}$ and $O_{FeFe-FeNb}$ sites of Fe_3_Nb_1_ and Fe_2_Nb_2_ terminations remains energetically favorable from 0–900 K, whereas the reactions at other sites, *i. e*. the *Fe−0*, *Nb−0*, $O_{FeFe-NbNb}$ , $O_{FeNb-FeNb}$ and $O_{NbNb-FeNb}$ sites, become endothermic processes as the temperature rises. In particular, we found that dissociation at the $O_{NbNb-FeNb}$ sites is significantly endothermic even at 0 K. On the Fe_1_Nb_3_ termination, the $O_{FeFe-FeNb}$ and $O_{FeFe-NbNb}$ are the two thermodynamically favorable sites between 0–900 K, with the dissociation energy ranging from −1.96 to −1.57 eV and −1.54 to −0.18 eV, respectively, whereas the reactions at the $O_{FeNb-FeNb}$ , $O_{NbNb-FeNb}$ , $O_{NbNb-NbNb}$ , *Fe−0* and *Nb−0* sites become endothermic from 900 K, 600 K, 600 K, 300 K and 0 K, respectively.

Overall, our simulations indicate that the dissociation energies on all the surfaces increase with temperature and the energies on the Fe_3_Nb_1_ and Fe_2_Nb_2_ terminations show the same order of E_NbNb−NbFe_ < E_Nb−0_<E_FeFe−NbNb_<E_FeNb−FeNb_<E_FeFe−FeNb_<_EFeFe−FeNb_, whereas the order changes into E_Nb−0_<E_Fe−0_<E_NbNb−NbNb_<_EFeNb−FeNb_<E_FeFe−NbNb_<E_FeFe−FeNb_ on the Fe_1_Nb_3_ termination. Interestingly, on the one hand we found that the number of Fe cations coordinated to the O sites can affect the dissociation energy. For instance, the H_2_ molecule prefers to be dissociated at the $O_{FeFe-FeFe}$ and $O_{FeFe-FeNb}$ sites, with respect to the $O_{NbNb-NbNb}$ and $O_{NbNb-NbFe}$ sites. On the other hand, the dissociation energies at the $O_{FeFe-FeFe}$ and $O_{FeFe-FeNb}$ sites of the three (010) surfaces are in agreement with the trend in the work functions, shown in Table 1.

**Figure 9:**
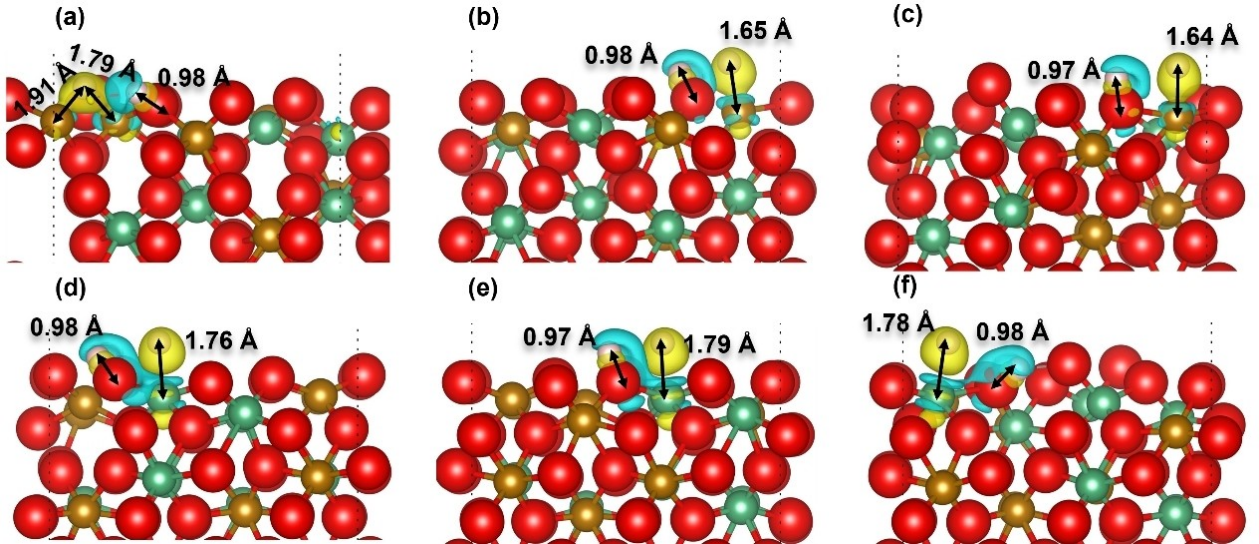
Charge transfer difference showing iso‐surface level of 0.05 eV/Å^3^ at the (a‐c) Fe−O and (d‐f) Nb−O sites of the (010) surfaces after dissociation of H_2_; (a, d) Fe_3_Nb_1_‐termination; (b, e) Fe_2_Nb_2_‐termination; (c, f) Fe_1_Nb_3_‐termination; (d) Fe_8_Nb_8_‐termination; O atoms are red; Fe atoms are yellow; Nb atoms are green; H atoms are white; yellow color represents charge depletion and blue color represents charge gain.

In previous work,[^44^, ^45^, ^46^, ^47^, ^48^] the dissociation of the hydrogen molecule at the O sites has been studied extensively and H atoms coordinated by oxygen are generally oxidized after dissociation. To explore how the electrons are transferred at the metal‐oxygen sites in this work, we have focused on the charge transfer and other properties, *i. e*. Bader charge and bond length, at the Fe/Nb−O sites, see Figure 9.

Our simulations suggest that the H atoms from the O−H and the Fe/Nb−H groups are surrounded by electron depletion and gain areas, respectively. Correspondingly, a relatively small gain and depletion area is distributed around the O and Fe/Nb atoms, respectively. We found that the electron depletion area around the hydroxyl at the Nb−O site is slightly larger than that at the Fe−O site. The calculated Bader charge of the H for the hydroxyl ranges from +0.60 to +0.67, whereas the H atoms coordinated by Fe have Bader charges of −0.19 to −0.29, which is smaller than those bound to Nb, see Table 2. In addition, we have shown the bond lengths in Figure 9, where the Fe−H bond at 1.79 Å is slightly longer than the Nb−O bond (1.76 Å) on the Fe_3_Nb_1_ terminations. In contrast, in the Fe_2_Nb_2_ and Fe_1_Nb_3_ terminations, the Nb−O bond length is longer than that of Fe−O, because on the Fe_3_Nb_1_ termination the Fe cation in the sub‐surface layer exhibits similar chemical activity towards the dissociated H atom, which therefore tends to move closer to this Fe atom at a distance of 1.91 Å after optimization. An O−H bond length of approximately 0.98 Å was measured in all the hydroxyl groups. Overall, our simulations demonstrate that after dissociation at the metal‐oxygen sites, one H atom becomes bonded tightly to the oxygen, forming a hydroxyl group, whereas another H atom is captured by the surrounding metal cations, forming the relatively weak Fe/Nb−H bond. In this process, electrons are transferred from one dissociated H atom to the coordinated O atom, whereas the other H atom obtains electron from the metal site.

**Table 2:** Bader charge (*q*) of the adsorbed H ions coordinated by Fe/Nb and O on the (010) surfaces.

	*q*_H_ (e)			
	Fe−O site		Nb−O site	
	Fe−H	O−H	Nb−H	O−H
Fe_3_Nb_1_‐termination	−0.21	+0.61	−0.32	+0.68
Fe_2_Nb_2_‐termination	−0.29	+0.64	−0.37	+0.67
Fe_1_Nb_3_‐termination	−0.19	+0.60	−0.37	+0.68

#### 2.2.2 (111) Surface

**Figure 10:**
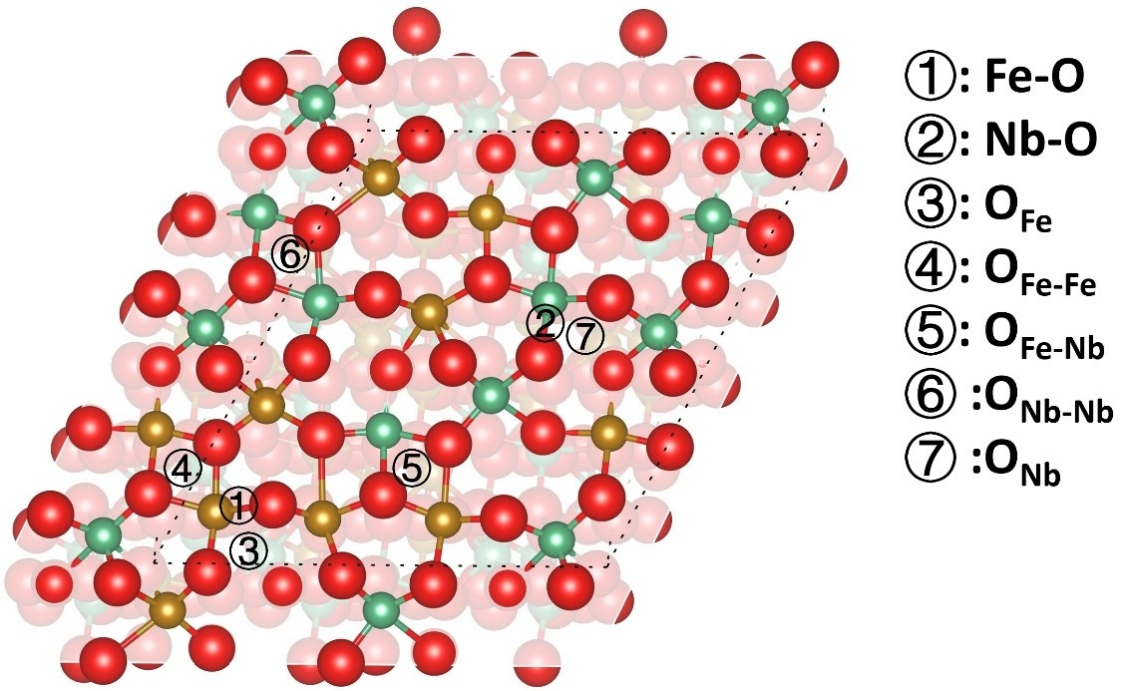
Dissociation sites of H_2_ on the (111) surface; O atoms are red; Fe atoms are yellow; Nb atoms are green.

On the (111) surface, we found that five‐fold cations are exposed in the top layers and we have therefore taken into account the seven adsorption sites, including two metal‐oxygen sites that are similar to the (010) surfaces, and five oxygen‐oxygen sites. For the oxygen‐oxygen sites on the (111) surface, $O_{Fe/Nb}$ denotes the site where the two oxygen are coordinated by one five‐fold cation, whereas in another type of reaction site, *i. e*. $O_{Fe-Fe}$ , $O_{Fe-Nb}$ and $O_{Nb-Nb}$ , the two oxygen atoms are shared by two Fe/NbO_5_ square pyramids, see Figure 10.

**Figure 11:**
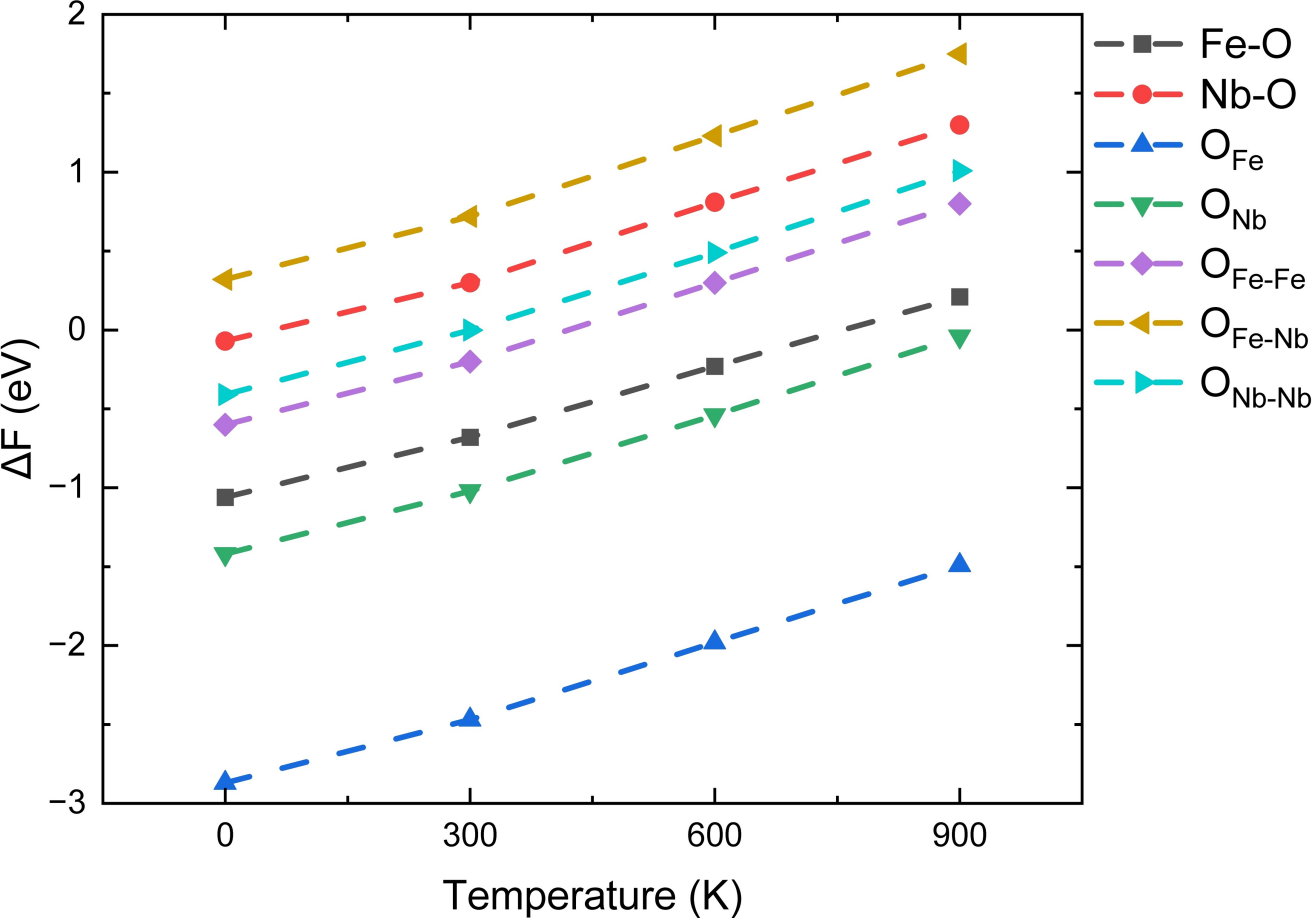
Dissociation energy of H_2_ on the F_8_N_8_‐terminated surface between 0–900 K.

Figure 11 shows the dissociation reaction energy at the (111) surface under different temperatures. We found that reactions at the $O_{Fe}$ site are thermodynamically much more favourable than at the other three sites, with the dissociation energies remaining negative from 0–900 K, and they are even larger than those found for W‐doped Al_2_O_3_ and WO_x_ materials.[^44^, ^45^] At another oxygen site, *i. e*. the $O_{Nb}$ site, the dissociation reaction is exothermic by −1.74 eV at 0 K but increases to ~0 eV when the temperature reaches 900 K. At the other sites, the reactions gradually become endothermic as the temperature increases, with the exception of the $O_{Fe-Nb}$ site, where the dissociation energy is positive from 0 K upwards.

In general, our simulations indicate that the $O_{Fe}$ site is the thermodynamically most favorable site on the (111) surface, and the priority of the reaction is the sequence: $E_{O_{Fe-Fe}}<E_{Nb-O}<E_{O_{Nb-Nb}}<E_{O_{Fe-Fe}}<E_{Fe-O}<E_{O_{Nb}}<E_{O_{Fe}}$ . In addition, we found that the oxygen sites on the (111) surface have similar chemical activity to those on the (010) surfaces, but the Fe/Nb−O sites on the (111) surface are more active than on the (010) surfaces, suggesting that the five‐fold cations exposed on the top layer play a more important role in the dissociation of H_2_ than the four‐fold cations.

**Figure 12:**
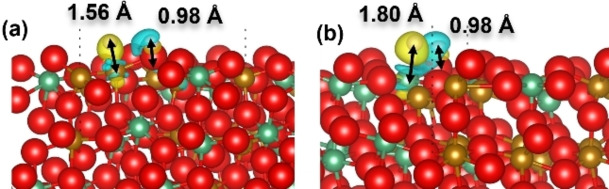
Charge transfer difference showing iso‐surface level of 0.05 eV/Å^3^ at the (a) Fe−O and (b) Nb−O sites of the (111) surface after dissociation of H_2_; O atoms are red; Fe atoms are yellow; Nb atoms are green; H atoms are white; yellow color represents charge depletion and blue color represents charge gain.

The charge transfer at the Fe/Nb−O sites in the form of charge density difference plots are shown in Figure 12. Our simulations suggest partial electronic density transfer from the cations to hydrogen at the Fe/Nb−O site and from hydrogen to oxygen at the O−H site, similar to the change of electronic distribution in the (010) surfaces. In addition, we found that the electron gain area around the Nb site is larger than that around the Fe site, in agreement with the larger Bader charge of −0.39 e for the H coordinated by Nb, see Table 3. The O−H bond lengths in the hydroxyl groups are close to 0.98 Å, whereas the Fe−H and Nb−H bonds at the Fe/Nb−O site are longer than the O−H by 0.58 and 0.82 Å, respectively. Compared with the (010) surfaces, we found that the differences in charge transfer, Bader charge and the bond length of the metal/oxygen‐H between the (010) and (111) surfaces are small, with the exception of the Fe−O site of the (111) surface where the H coordinated by Fe has the smallest Bader charge of −0.16 e and the Fe−H has the shortest bond length at 1.56 Å.

**Table 3:** Bader charge (*q*) of the adsorbed H ions coordinated by Fe/Nb and O on the (111) surface.

	*q*_H_ (e)			
	Fe−O site		Nb−O site	
	Fe−H	O−H	Nb−H	O−H
Fe_8_Nb_8_‐termination	−0.16	+0.62	−0.39	+0.63

### 2.3 Formation of Water

**Figure 13:**
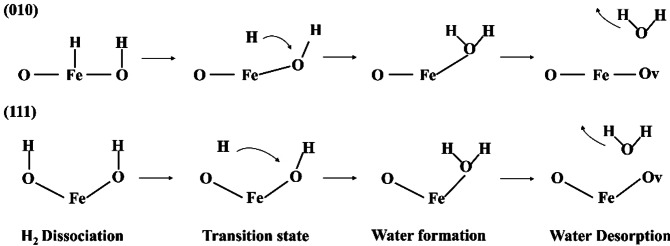
Schemes of pathways of the water formation reaction on the (010) surface (top) and the (111) surface (bottom); O and Fe indicate the atoms in the top surface layers, whereas Ov refers to an oxygen vacancy.

The dissociation of hydrogen at the surfaces revealed above shows that the formation of water could take place at both the Fe/Nb−O and O−O sites. To gain insight into the mechanisms, we have chosen the Fe−O sites of the (010) surfaces and the $O_{Fe}$ site of the (111) surface to simulate the migration pathways of dissociated H atoms and the water formation reactions. After dissociation on the (010) surfaces, the H atoms coordinated to the Fe cations migrate towards the nearby oxygen ions to form a H_2_O molecule, which then desorbs from the surfaces, forming an oxygen vacancy on the top layer, which in the SOFC process will be filled again by the oxygen ions from the electrolyte. The proposed reaction process is shown in Figure 13.

**Figure 14:**
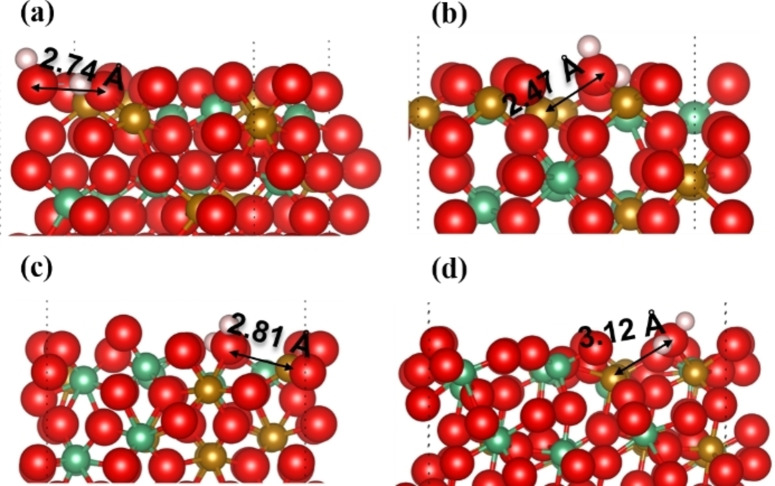
Optimized structures after the diffusion of H atoms on; (a) Fe−O site of the Fe_3_Nb_1_‐terminated (010) surface; (b) Fe−O site of the Fe_2_Nb_2_‐terminated (010) surface; (c) Fe−O site of the Fe_1_Nb_3_‐terminated (010) surface ;(d) O_Fe_ site of the Fe_8_Nb_8_‐terminated (111) surface; O atoms are red; Fe atoms are yellow; Nb atoms are green; H atoms are white.

Figure 14 shows the hydrogen migration at the surfaces after dissociation. Our simulations show that the H_2_O molecule is not stable on the Fe_3_Nb_1_ termination. Instead, one H atom tends to be captured by the adjacent oxygen ion, forming a hydrogen‐bond, leading to the two top O ions forming the hydroxyl groups getting close to each other within a distance of 2.7 Å. In contrast, we found that the H_2_O molecules are stable on both the Fe_2_Nb_2_ and Fe_1_Nb_3_ terminations, where the bond length of the nearby metal‐oxygen on the Fe_1_Nb_3_ termination is longer by 0.3 Å compared to the Fe_2_Nb_2_ termination. In addition, we found that the longest bond distance of 3.12 Å is the (H)O(H)−Fe bond on the Fe_8_Nb_8_ (111) surface.

Overall, our calculations suggest that the surface‐bound H_2_O molecules, formed after dissociation of the H_2_ molecules, are generally stable at the surfaces, with the exception of the Fe_3_Nb_1_ termination. These water molecules could leave the surfaces, as evidenced by the lengthening of the metal‐oxygen bonds compared to those in the bulk structure.^[42]^ We would suggest that the H atoms on the Fe_3_Nb_1_ termination (Fe‐rich) preferring to form two hydroxyl groups could be partially due to the oxygen ions in the Fe‐rich region having a similar chemical activity towards the dissociated H atoms.

**Figure 15:**
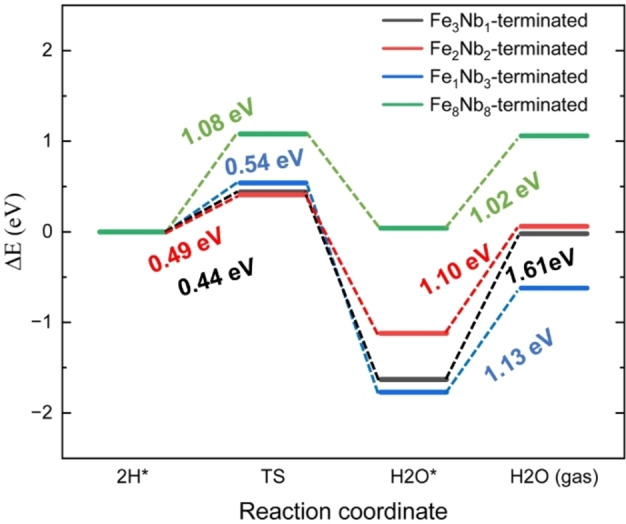
Energy pathways of diffusion of H and water formation on the four surfaces; TS refers to the transition state from H_2_ dissociation to H_2_O formation; * refers to the surface‐bound state, and the energies shown for the two steps refer to the energy barriers from 2H* to TS and H_2_O* to H_2_O (gas), respectively. The energies in the left represent activation energies to the TS, whereas the numbers in the right represent desorption energies of the water leaving the surface.

The energy pathways are plotted in Figure 15. Our calculations show that the energy barriers from the dissociated *2H to the adsorbed H_2_O* states (* indicates a surface‐bound species) are in the range of 0.44–0.54 eV on the three (010) surfaces, compared to 1.08 eV on the Fe_8_Nb_8_‐terminated (111) surface, which is still much lower than the migration barrier of 3.53 eV reported for the pure CeO_2_ system.^[46]^ In addition, the formation of the surface‐bound water molecules is an energetically favourable process on the (010) surfaces, although on the (111) surface, the energy of the H_2_O* state is similar to that of the *2H state. Finally, the desorption from the surfaces into a gaseous water molecule is also calculated, which is represented by the process from the H_2_O* to the H_2_O(g) state. The lowest barrier (1.02 eV) is found on the (111) surface, which is larger than the barrier found at the Ni‐YSZ anode (~0.6 eV), but close to that found for the CeO_2_ material (~0.9 eV),[^46^, ^47^, ^48^] indicating that the FeNbO_4_ phase exhibits catalytic properties that are compatible to those two commonly used SOFC anode materials, where the dissociation of hydrogen is concerned.

Overall, our simulations indicate that migration of the dissociated H atoms on the (111) surface needs to overcome a larger barrier than on the (010) surfaces to form the surface‐bound water molecules, which can be explained by the fact that the O−H bond is stronger than the Fe−O, in agreement with the analysis of the Bader charges. In addition, we found that water formation on the Fe_3_Nb_1_ termination is more endothermic than on other surfaces, indicating that forming gaseous water molecules through two hydroxyl groups is energetically difficult compared to the directly bonded water molecules.

## 3 Conclusions

We have employed DFT+U‐D2 calculations to investigate the surface properties of the orthorhombic FeNbO_4_ material, the adsorption and dissociation of H_2_ molecules, the migration of H ions, as well as the formation of water at the (010) and (111) surfaces. First, the calculations of the surface properties have demonstrated that the five‐fold cations in the (111) surface show a higher oxidation state than the four‐fold cations in the (010) surfaces, and the bandgaps of those surfaces are reduced to less than 0.5 eV. The interplanar relaxations indicate that the first two top layers of the three (010) surfaces move in opposite directions, leading to the metal cations in the sub‐surface layers becoming exposed to the vacuum, whereas the relaxed two layers of the (111) surface shift outwards from the bulk. We have selected the Fe/Nb−O and O−O sites on the (010) and (111) surfaces as the dissociation sites for the H_2_ molecule. At the Fe/Nb−O sites, the five‐fold cations of the (111) surface enable dissociation more readily than the four‐fold Fe/Nb in the (010) surfaces. In addition, our calculations indicate that the numbers of Fe cations coordinated to the O can affect the dissociation energies. Specifically, dissociation at the $O_{FeFe-FeFe}$ and $O_{FeFe-FeNb}$ sites of the (010) surfaces remains exothermic from 0–900 K, whereas the reaction at other sites becomes endothermic at temperatures below 900 K. Similarly, we found that the hydrogen molecule prefers to dissociate at the $O_{Fe}$ site of the (111) surface, where the reactions remain exothermic in the range of 0–900 K. Charge transfer investigations at the Fe/Nb−O sites have shown that on both the (010) and (111) surfaces the H atoms obtain electron density from Fe/Nb, whereas the electrons are transferred from the other dissociated H atom to the coordinated O atom.

Finally, we have simulated the migration of H ions and the formation of water at the Fe−O sites in the (010) surface and the $O_{Fe}$ site of the (111) surface. The barriers for the diffusion of H ions range from 0.44–0.54 eV on the three (010) surfaces, but t is higher at 1.08 eV on the (111) surface. The lowest calculated barrier for the formation of surface‐bound water is found for the $O_{Fe}$ site of the (111) surface at 1.02 eV, which is close to that of other anode materials, e. g. Ni‐YSZ and CeO_2_.

Overall, our simulations suggest that the oxygen bridge sites both on the (010) and (111) surfaces display more chemical reactivity towards H_2_ dissociation than the metal‐oxygen bridge sites. However, the diffusion of dissociated H ions from the oxygen bridge sites is energetically less favorable than from the Fe/Nb−O sites.

We consider that this work, which has revealed the mechanisms of hydrogen dissociation and water formation at individual sites on two major surfaces of disordered o‐FeNbO_4_, shows that this material has promising characteristics for SOFC anode applications. For example, our findings have revealed that FeNbO₄ offers several thermodynamic and kinetic advantages: (i) the relatively low energy barriers for hydrogen dissociation and water formation on key surfaces, (ii) the stability of the surface‐bound H atoms in the form of hydroxyl groups, and (iii) the low bandgap values at the surfaces, which indicate enhanced electronic conductivity. In addition, the five‐fold coordination of Fe and Nb on the (111) surface significantly improves the dissociation of H₂, indicating strong reactivity at these sites. We consider that these features position FeNbO₄ as a potential alternative to Ni‐based anodes. We trust that our study will provide guidance for future experimental work.

## Conflict of Interests

The authors have no conflict of interest to declare.

4

## Data Availability

All data created during this research are provided in full in the results section of this paper.
